# Evolution of Chemical Composition and Modeling of Growth Nonmetallic Inclusions in Steel Containing Yttrium

**DOI:** 10.3390/ma14237113

**Published:** 2021-11-23

**Authors:** Dorota Kalisz, Paweł L. Żak, Sergey Semiryagin, Sergey Gerasin

**Affiliations:** 1 Faculty of Foundry Engineering, AGH-University of Science and Technology, al. A. Mickiewicza 30, 30-059 Krakow, Poland; pawelzak@agh.edu.pl; 2 LLC Technical University Metinvest Polytechnic, 71a Sechenova Str., 87524 Mariupol, Ukraine; nipkidongtu@gmail.com (S.S.); sergii.gerasin@gmail.com (S.G.)

**Keywords:** steel, refining, non-metallic inclusions, yttrium, computer simulation

## Abstract

The programs WYK_Stal and Bi-Growth, developed at AGH-UST, Kraków, Poland, were used for simulating the refining process, the formation of non-metallic inclusions, and their growth. The Fe-Y-Al-O-S-Ca system in pre-oxidized steel was analyzed, where yttrium formed precipitates from both O and S. When first Al and second Y were added to steel, the proportion of Al_2_O_3_ inclusions remained constant. This resulted in higher yttrium losses for oxide formation, whereas the sulfur content promoted sulfide phase formation. The introduction of yttrium at the end of refining contributed to reducing the consumption of this element in the non-metallic phase formation. The addition of aluminum and then calcium were sufficient to achieve a high degree of deoxidation and desulfurization. Calculations performed with WYK_Stal for both (a) and (c) versions of the model showed that the sulfide phase was constituted by CaS and FeS (model c) and CaS (model (a)). The participation of the calcium sulfide phase turned out to be dominant in the inclusions. Their presence was also identified in the slag phase. Simulations of the growth of complex oxide and oxo-sulfide inclusions using the Bi_Growth program showed that the yttrium content of the steel has a decisive role in the formation of complex oxide inclusions and the final oxygen content of the steel. In contrast, for the growth of oxide-sulfide inclusions, the character of growth is determined by the sulfur content of steel.

## 1. Introduction

During liquid steel deoxidation, non-metallic inclusions are formed and they are a mixture of different oxides. After Si and Mn deoxidation, liquid inclusions SiO_2_–MnO are formed, and the Al admixture favors the mixed type inclusions formation, with the dominance of pure Al_2_O_3_ [1,2,3,4,5,6]. Non-metallic inclusions formed during refining rarely occur in the form of simple precipitates; more often they appear as complex precipitates or mixtures of simple compounds. The Al_2_O_3_, CaO, and SiO_2_ inclusions are detrimental to steel, therefore modifications or treatments are necessary to eliminate these unfavorable precipitates of both oxides (mainly Al_2_O_3_), as well as manganese sulfides by the addition of components with high chemical affinity to sulfur and oxygen, such as calcium, rare earth metals, and yttrium. 

Calcium is introduced to steel as CaSi powder, CaSiBa powder, CaSi, and AlCa. The process is mainly based on the transformation of solid inclusions Al_2_O_3_ into calcium aluminates, which remain liquid at the steel casting temperature. The prerequisite of liquid aluminates formation is the presence of CaO in the inclusions ranging from 40% to 60%, which corresponds to 3CaO∙Al_2_O_3_, 12CaO∙7Al_2_O_3_, and CaO∙Al_2_O_3_. The CaO participation in the inclusions will depend on the O, S, Al, Mn, S, and Si content in liquid steel. The formation of CaS precipitates is a consequence of the high chemical affinity of calcium to sulfur. At higher sulfur content, the simultaneous formation of MnS and CaS sulfides is possible; a higher sulfur content will increase the amount of MnS inclusions and also promote the formation of complex precipitates (Mn,Ca)S. In the case of sulfides, mutual solubility of MnS and CaS is observed, so that complex (Ca,Mn)S precipitates are formed. It was found out that the degree of modification of manganese sulfides with calcium depends on Ca/S and Ca/O ratios [4,6,7,8,9,10,11]. Research conducted by [11,12,13,14] showed that for providing optimal modification, the atomic concentration ratio ACR = 32 (wt.% Ca)/40 (wt.% S) > 1.8, if (wt.% Ca)/(wt.% S) < 0.32 (ACR, 0.4) the number of unmodified MnS sulfides increases. However, the optimal Ca addition affecting the Ca/S content varies considerably depending on the content of oxygen dissolved in steel. Modification of inclusions with Ca has some technological limitations due to the high vapor pressure (1.8 MPa at 1600 °C) and low solubility [7]. Therefore, it is reasonable to look for solutions in which Ca can be replaced with another material. As an alternative, zirconium, barium, or rare earth elements (lanthanum, cerium, praseodymium, neodymium, yttrium) can be added in the form of mischmetal, preliminary alloys, or pure metal (yttrium) in the process of non-metallic inclusions modification [8,9,10,11,12,13,14,15,16,17,18,19].

Yttrium is a metal with a number of unique properties, hence it is used for manufacturing both ferrous alloys as well as non-ferrous alloys and ceramics [20,21,22]. In ferrous alloys, yttrium is a highly active metal showing high chemical affinity, especially to oxygen, hence in a liquid metal bath it can act as a strong deoxidant. Reacting with elements dissolved in a liquid metal bath it forms solid, crystalline precipitates of oxides, but also sulfides, oxosulfides, nitrides, carbides, and carbide nitrides [20]. Minor oxide precipitates, especially Y_2_O_3_, act as a finely dispersed and also play the role of crystallization and nucleation centers for nitrides and carbides. During heat treatment, some unstable sulfides may dissolve and deform. This unfavorable phenomenon can be eliminated by adding yttrium and bonding it, for example, in Y_2_O_2_S. This compound does not dissolve, thus preventing the segregation of sulfur at grain interface, additionally causing ‘precipitate strengthening’. In contrast, the elimination of MnS inclusions in favor of hard sulfides Y_2_S_3_ is done through refining and separation processes occurring during solidification. This effect can be achieved by maintaining a certain level of oxygen and sulfur in the liquid metal bath and by forming non-metallic inclusions having well-defined chemical composition and comminution [23,24].

The thermodynamic analysis of non-metallic inclusions formation after adding yttrium to liquid steel requires determining phase systems that define the stability of the formed compounds. The presence of Y_2_О_3_, Y_2_О_2_S, YS, YN, YC_2_, and YS, which are the products of deoxygenation, desulfurization, denitrification, and decarburization, was identified [21,23,24,25]. The equilibrium constants of the noted reactions cited after W. Longmei and others [21] are presented in Table 1.

Based on the data in Table 1, the phase equilibrium system of Fe-Y-S-O was determined for 1600 °C. The diagram shows that inclusions are the first to form through deoxidation, therefore Y_2_O_3_ should be the first compound to precipitate. Their formation takes place simultaneously with the formation of oxosulfides Y_2_O_2_S [25]. Yttrium sulfide phase YS is formed with higher yttrium activity, lower sulfur activity, and lowest oxygen activity. The modification with yttrium results in a complete desulfurization with a strict control of Y/S levels. At the same time, the MnS formation is limited with Y-sulfur compounds. However, to provide complete modification, the ratio Y/S > 3 has to be maintained [25].

Therefore, the process of modification and introduction of additives cannot be considered in isolation, as their effects are complex and complementary. The change in the chemical composition of non-metallic inclusions due to the introduction of Al, Y, Ca additives was studied using non-commercial computer programs WYK_Stal and Bi-Growth.

## 2. Materials and Methods

In the process of liquid metal bath refining, the resulting non-metallic inclusions are heterogeneous and create a mixture of different oxides or oxides and sulfides. These compounds are a consequence of the introduction of metals and ferroalloys in the form of complex deoxidants, resulting in the formation of a liquid or solid-liquid non-metallic phase. The function of the deoxidant in steel is usually played by silicon, manganese, and aluminium, and therefore it is possible to form a liquid phase containing their oxides and also iron oxide [23,24,26,27,28,29,30,31,32,33,34,35].

### 2.1. Calculation of Non-Metallic Phase Formation with Liquid Steel in Thermodynamic Equilibrium Conditions (WYK_STAL)

The formation of oxide and sulfide non-metallic inclusions in liquid steel was simulated with the WYK_STAL computer program. It is used to support steelmaking processes in real time and was developed at the AGH University of Science and Technology, Department of Physical and Chemical Foundations of Metallurgy, Faculty of Metals Engineering and Industrial Computer Science. The program can be also used for simulating steel refining processes and introducing alloying additives into steel prior to the casting process on a continuous casting line (CCS). The block diagram of the program is presented in papers [26,28]. The program has internal databases, physicochemical parameters for steel and slag, and consists of several modules supporting a specific process [26,28]. The principle of the WYK_STAL program was presented in papers [24,26,27,28,29,30]. Calculations of non-metallic inclusions formation can be performed in several variants offering appropriate models considering the physicochemical properties of the non-metallic inclusions: assuming the activity of the formed compound a = 1 (model a), the activity coefficient of metal components f = 1 (model b), and the metal-slag interfacial partition coefficient (model c) (Table 2). To evaluate the stability of yttrium compound formation in steel and the interaction with other precipitates, it is necessary to identify the possible compounds to be formed in the presence of yttrium. Data on double and triple phase systems containing yttrium oxide are limited to diagrams typical of steel metallurgy: Y_2_O_3_–CaO, Y_2_O_3_–FeO and Y_2_O_3_–Al_2_O_3_. Phase diagrams for CaO–Y_2_O_3_, Al_2_O_3_–Y_2_O_3_, FeO–Y_2_O_3_, FeO–Al_2_O_3_–Y_2_O_3_ and FeO–CaO–Y_2_O_3_ were determined by G.G Mikhailov and others and published in [31].

The analysis of those phase systems reveals that solid precipitates (Y_2_O_3_, 3Y_2_O_3_·5Al_2_O_3_, Al_2_O_3_) and liquid oxide phases may be the products of deoxidation when yttrium and aluminum are introduced to the steel at 1600 °С. In contrast, the addition of calcium and yttrium results in the formation of phases Y_2_O_3_, 3CaO·2Y_2_O_3_, CaO·Y_2_O_3_, CaO·2Y_2_O_3_ and the corresponding liquid oxide phases.

Calculations have been performed under thermodynamic equilibrium conditions for steel with the chemical composition shown in Table 3.

The following data were assumed for the process simulation: process time: 30 min; ladle metal mass: 140,000 kg; slag mass: 100 kg; metal temperature at the beginning of the process: 1670 °C; gas phase pressure: 1 atm; initial maximum oxygen content bound in inclusions: 0.0001%; initial total oxygen content in steel: 0.01%; initial slag composition: CaO—45%; Al_2_O_3_—2%; MgO—9%; MnO—5%; SiO_2_—12%; FeO—27%; change of additives dosed to slag: 100 kg CaO was added in the first minute and 100 kg SiO_2_ in second minute. The refining process was analyzed for the system Fe-O-S-Al-Y-Ca. Calcium was introduced to modify the alumina inclusions and for possible desulfurization of the liquid metal bath. The additives were introduced to steel in the following order: 30 kg of aluminum was introduced at 1 min, 76 kg of yttrium at 10 min, and 20 kg of calcium at 20 min. Calculations were carried out for model variants a, b, c. The same procedure was the repeated, although yttrium was introduced after calcium with the same quantitative parameters.

### 2.2. Bi-Component Nucleus Growth Model (Bi-Growth)

Different ways of modeling particle growth are possible. In all cases, a spherical particle shape, stationary diffusion, and independent growth of precipitates are assumed. If the course of the growth process is determined by the diffusion of reactants to the reaction site, we speak of a ‘diffusion control’; if the process is controlled by a chemical reaction we are dealing with a chemical control model of the process, these cases being extreme. The current work proposes a solution to the bi-growth of oxide and sulfide precipitates by focusing on the formation of precipitates after introducing yttrium and aluminum to steel. Because high mixing energies are used during refining, the role of reactant diffusion into the reaction zone was neglected, and the system was treated as perfectly homogeneous in terms of chemical composition. Each precipitate was assumed to grow only on its surface, e.g., yttrium oxide grows only on the surface of Y_2_O_3_, and aluminum oxide on the surface of Al_2_O_3_. In this case, the particle growth rate was found to depend solely on the concentration of all three components (Y, Al, O). It was assumed that the nucleus consists of *n*_1_ moles of oxide 1 and *n*_2_ moles of oxide 2. Coefficient *Aij* is a quantity dependent on the supersaturation defined by the difference between the current and equilibrium oxygen concentration in steel and the reaction rate constant *kij*. An assumption was made that *Aij* > 0, which means that a deficiency in any of the components terminates the growth process of a given precipitate, but neither terminates the growth process of the entire precipitate, nor the dissolution of the particles [23]:

(1) $$A_{ij}=k_{ij}\times S_{i}^{\alpha_{i}}=k_{ij}\cdot {\left(c_{\left[0\right]}(t)-c_{\left[0\right]}^{\left(i\right)}\right)}^{\alpha_{i}}$$

where:

*A_ij_*—coefficient (depends on oxygen concentration),

*S_i_*—supersaturation,

α—order of reactions in the calculation (assumed = 1),

*k_ij_*—rate constant, depends on the oxygen “*i*” and surface “*j*”.

Hence the quantitative increase in oxide 1 and oxide 2 in the bi-component nucleus is:

(2) $$dn_{i}/dt=\left(A_{i1}\times n_{1}+A_{i2}\times n_{2}\right)/r^{\beta }$$

where: i=1,2, *β* = 1,

Hence:

(3) $$\frac{dn_{1}}{dt}=\frac{A_{11}n_{1}+A_{12}n_{2}}{r}$$

(4) $$\frac{dn_{2}}{dt}=\frac{A_{21}\times n_{1}+A_{22}\times n_{2}}{r}$$

*β* = 1,

*n*_1_—moles of oxide 1,

*n*_2_—moles of oxide 2,

*A_i_*_1_—coefficient dependent on oxide concentration (for oxide 1),

*A_i_*_2_—coefficient dependent on oxide concentration (for oxide 2).

The radius of a growing nucleus can be calculated with the equation:

(5) $$\frac{4}{3}\times \pi \times r^{3}=\sum_{i=1}^{2}\nu_{i}\times n_{i}$$

*r*—radius of nucleus [m],

*v*—molar volume [cm^3^·mole^−1^].

(6) $$r=\sqrt[{3}]{\frac{\frac{3}{4}\times \frac{1}{\pi }\times \left(\nu_{1}\times n_{1}+\nu_{2}\times n_{2}\right)}{z}}$$

*v*_1_—molar volume of oxide 1 [cm^3^·mole^−1^],

*v*_2_—molar volume of oxide 2 [cm^3^·mole^−1^].

The molar volumes of examples of non-metallic inclusions are shown in Table 4:

The growth of the inclusions results in a change in its chemical composition as expressed by the molar fraction of oxide 1 − *x*_1_.

(7) $$x_{1}=\frac{n_{1}}{n_{1}+n_{2}}$$

*m*_0_ = *m*_0_ − Δ*m*_0_—oxide increment;

(8) $$m_{Al}=m_{Al}-\Delta m_{Al}$$

(9) $$m_{Y}=m_{Y}-\Delta m_{Y}$$

**Figure 1:**
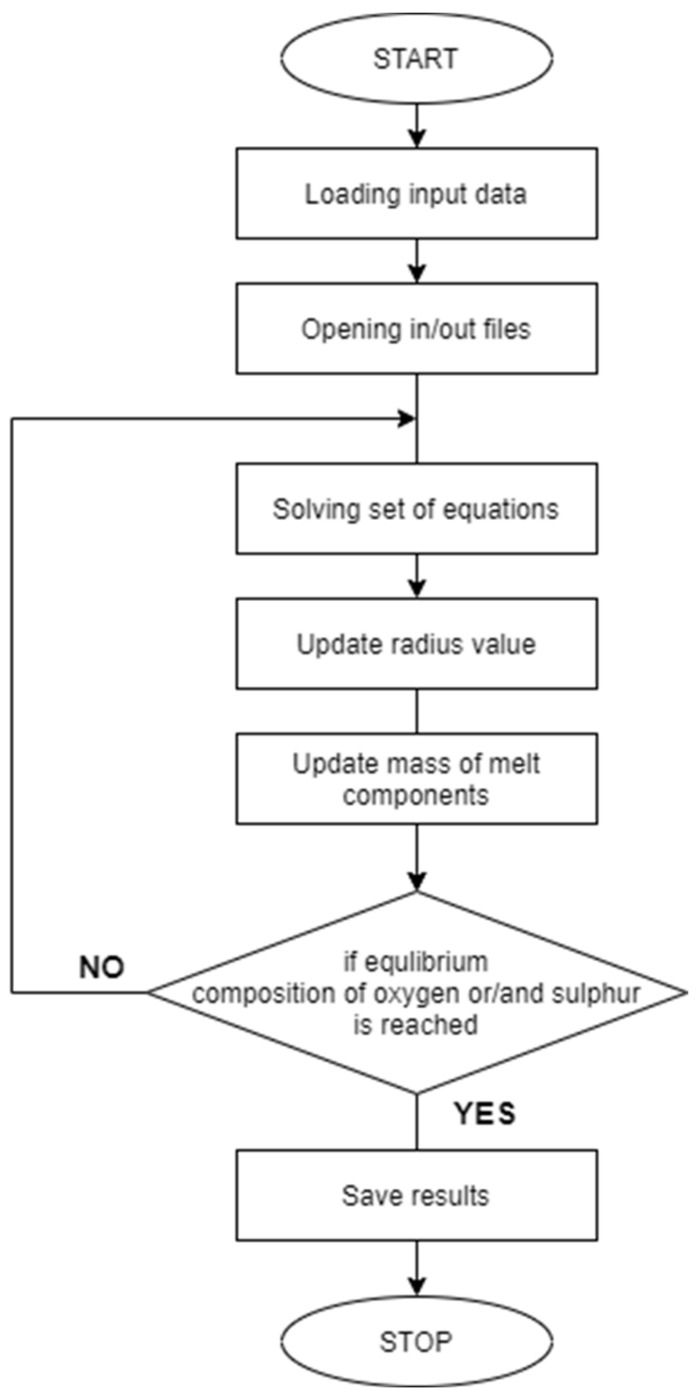
Block diagram of a program to calculate the Bi-growth of non-metallic inclusions during ladle refining of steel.

The block diagram of the computer program is shown in Figure 1.

## 3. Simulation Results

### 3.1. Simulation Results Obtained with the Computer Program WYK_STAL

**Figure 2:**
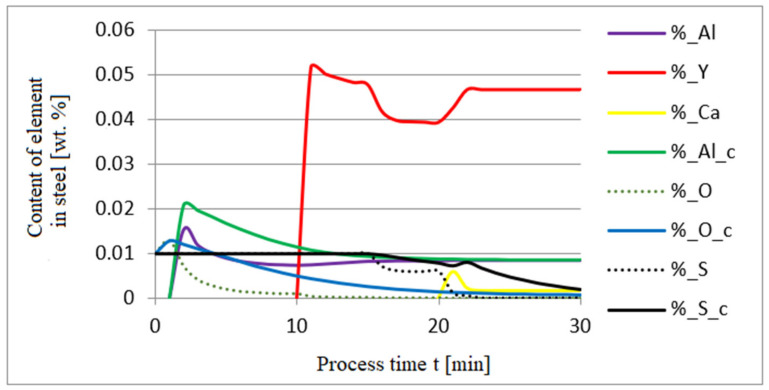
Change in the content of an element in liquid steel (wt.%) depending on the process time t (min).

**Figure 3:**
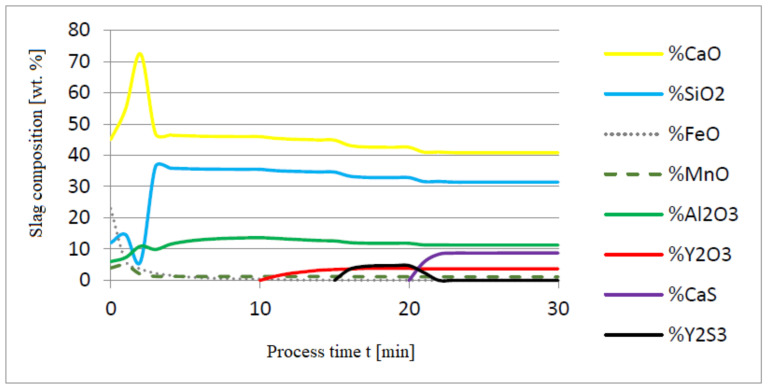
Change in chemical composition of slag (wt.%) depending on the process time t (min).

**Figure 4:**
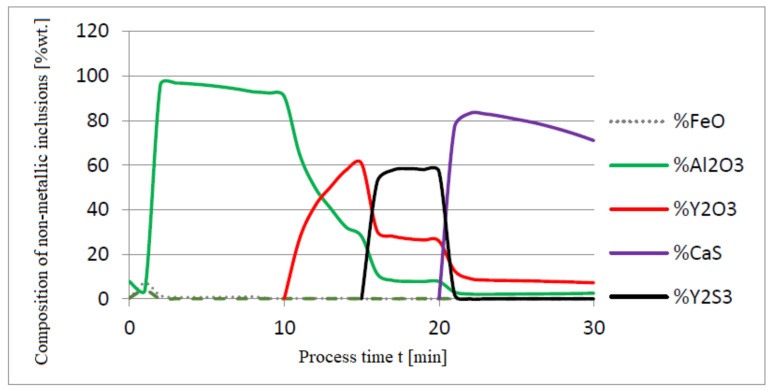
Change in chemical composition of non-metallic inclusions (wt.%) depending on the process time.

The additives were introduced to the steel in the following order: 30 kg of aluminum was introduced in the first minute, 76 kg of yttrium at 10 min time, and 20 kg of calcium at 20 min. Calculations were performed according to model a (non-metallic inclusion activity a = 1). The simulation results of the refining process using model (a) are shown in Figure 2, Figure 3 and Figure 4.

Initial deoxidation with aluminium resulted in the oxygen reduction by about 0.005% O, with a further addition of yttrium at 10 min resulting in deep deoxidation. The resulting drop in oxygen content resulted in yttrium consumption for sulphide formation. In addition, the introduction of calcium at the end of the process resulted in very strong desulfurization of the steel. As a result of these processes, the chemical composition of both the non-metallic inclusion phase and the slag phase changed. In the case of the slag phase, the presence of both oxides and sulfides was identified. The sulfide phase was enriched with yttrium and calcium sulfide. Special attention should be paid to the results of calculations obtained for the non-metallic inclusion phase, the evolution of the composition being a consequence of the deoxidizing and desulfurizing abilities of the applied alloying additives.

**Figure 5:**
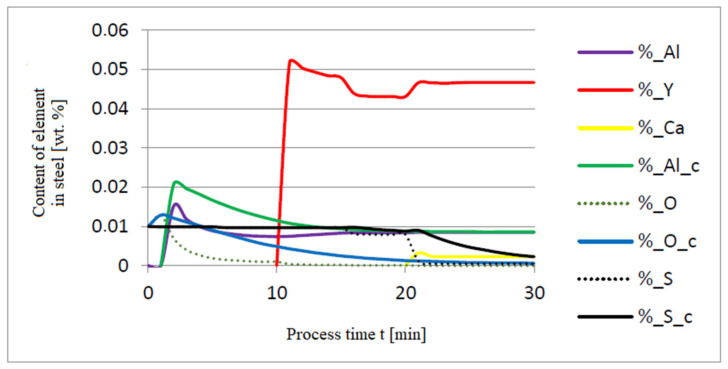
Change in the content of an element in liquid steel (wt.%) depending on the process time t (min).

**Figure 6:**
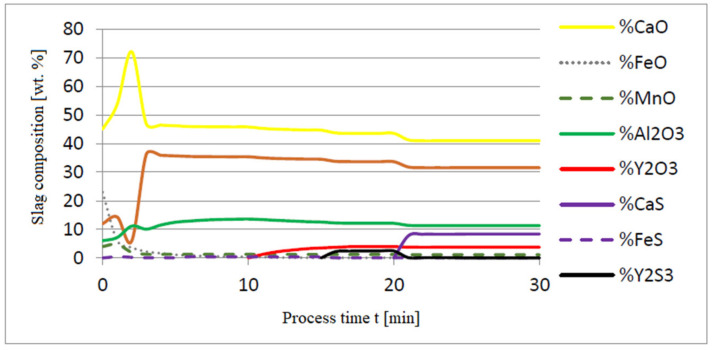
Change in chemical composition of slag (wt.%) depending on the process time t (min).

**Figure 7:**
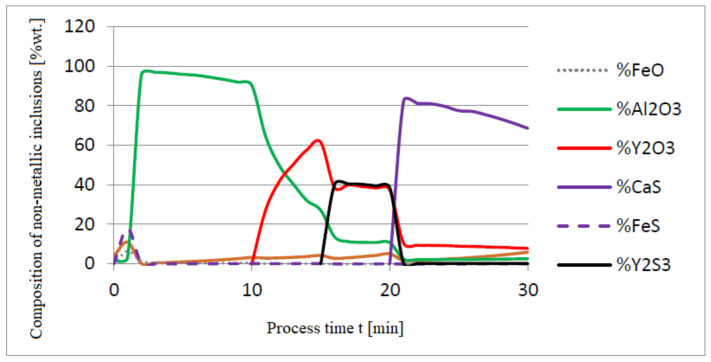
Chemical composition of non-metallic inclusions (wt.%) depending on the process time.

Figure 5, Figure 6 and Figure 7 show a series of calculations performed according to model (c). The obtained results of computer simulations are analogous to the results of calculations obtained for model (a).

**Figure 8:**
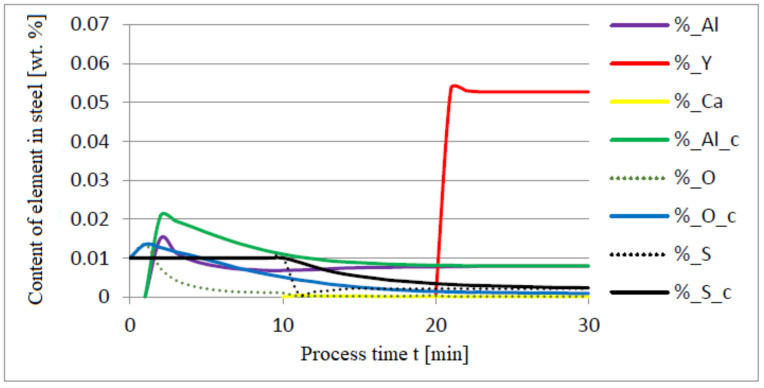
Change in the content of an element in liquid steel (wt.%) depending on the process time t (min).

**Figure 9:**
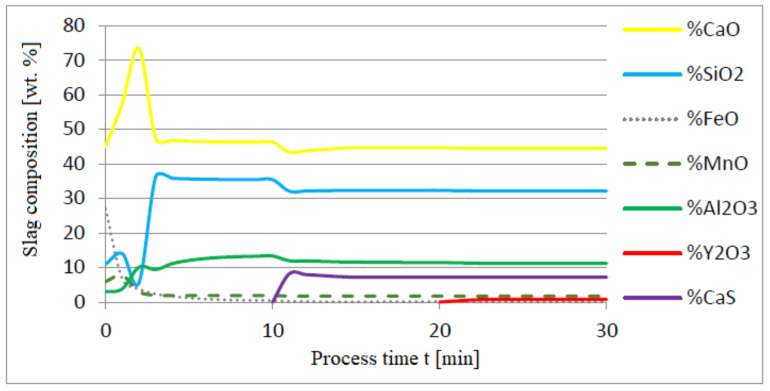
Change in chemical composition of slag (wt.%) depending on the process time (min).

**Figure 10:**
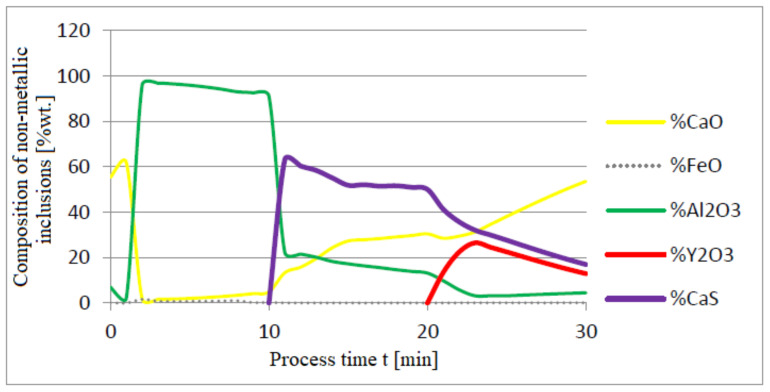
Change in chemical composition of non-metallic inclusions (wt.%) depending on the process time.

**Figure 11:**
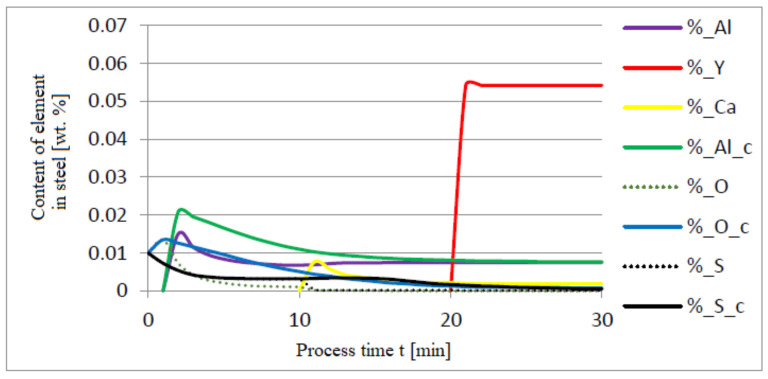
Change in the content of an element in liquid steel (wt.%) depending on the process time t (min).

**Figure 12:**
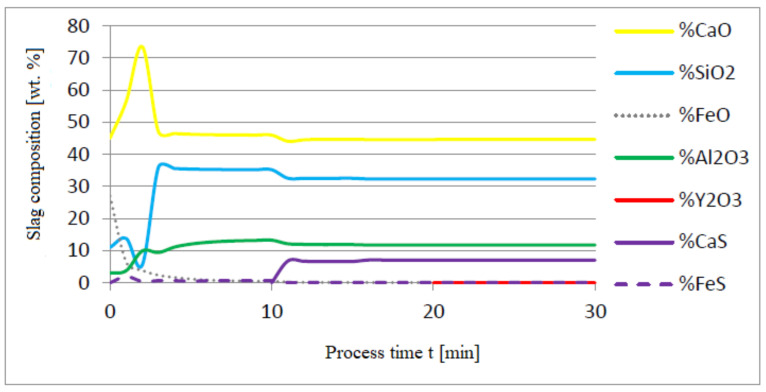
Change in chemical composition of slag (wt.%) depending on the process time t (min).

**Figure 13:**
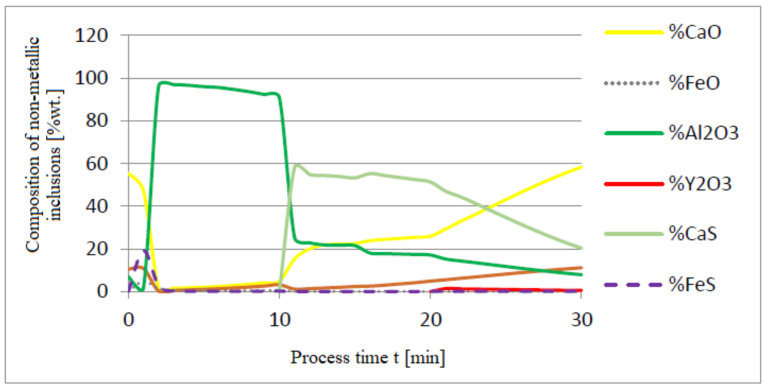
Change in chemical composition of non-metallic inclusions (wt.%) depending on the process time.

The following figures present the results of calculations in which the same quantities of additives were used, only the order of their dosing was changed: 30 kg of aluminium was added in the first minute of the process; after preliminary deoxidation, 30 kg of calcium was introduced in the tenth minute of the process, and 76 kg of yttrium in the twentieth minute. The results of calculations using model (a) are shown in Figure 8, Figure 9, Figure 10, Figure 11, Figure 12 and Figure 13.

The introduction of yttrium at the end of the refining process helps reduce the consumption of this element for the formation of the non-metallic phase. Moreover, the addition of aluminum and then calcium suffices to achieve a high degree of deoxidation and desulfurization of the liquid metal bath. Calculations performed for both a and c versions of the model showed that CaS and FeS constitute the sulfide phase (model c) and CaS (model a). The contribution of the calcium sulfide phase was dominant in the inclusion phase. Their presence was also identified in the slag phase.

### 3.2. Results of Calculations of the Growth of Bi-Component Non-Metallic Inclusions in Liquid Steel

**Figure 14:**
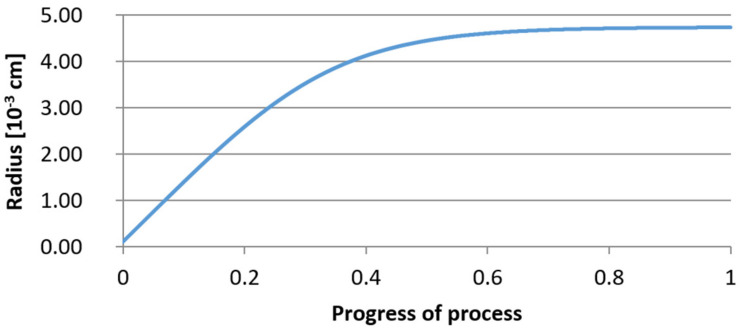
Increase of nucleus radius (initial radius r_0_ = 1 µm) in steel 1.

**Figure 15:**
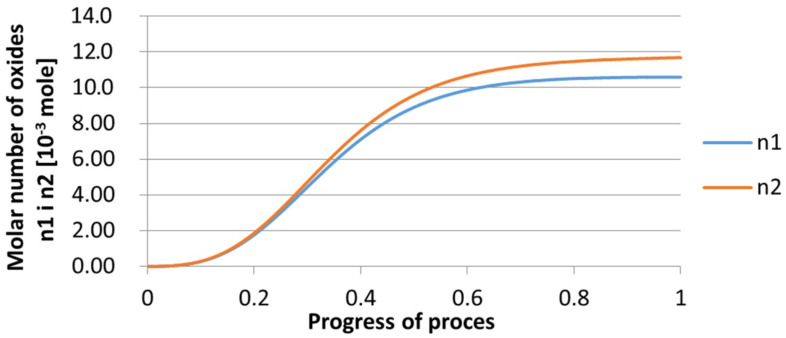
Increase of bi-component nuclei of oxides *n*_1_(Al_2_O_3_) and *n*_2_ (Y_2_O_3_) in steel 1 (for *r*_0_ = 1 µm).

**Figure 16:**
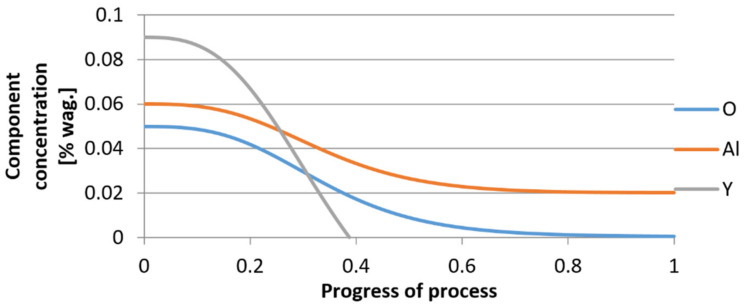
Change in component concentration during bi-component nucleus growth (Al_2_O_3_–Y_2_O_3_) in steel 1 (for *r*_0_ = 1 µm).

**Figure 17:**
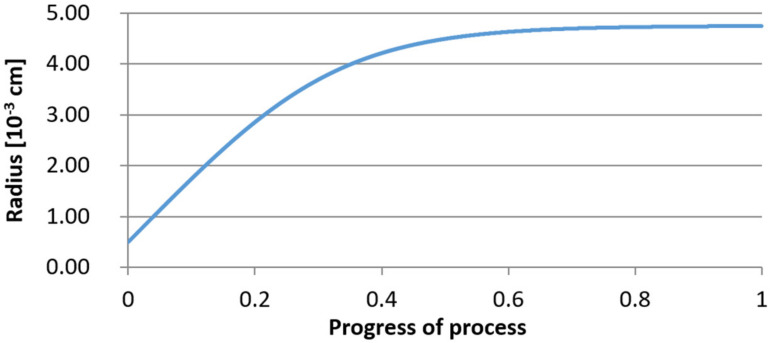
Increase of nucleus radius (with initial radius *r*_0_ = 5 µm) in steel 1.

**Figure 18:**
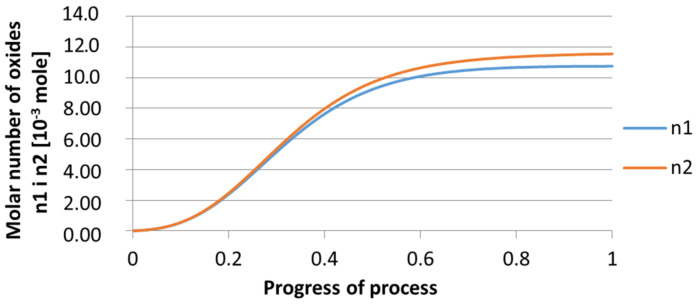
Growth of bi-component nuclei of oxides *n*_1_ (Al_2_O_3_) and *n*_2_ (Y_2_O_3_) in steel 1 (for *r*_0_ = 5 µm).

**Figure 19:**
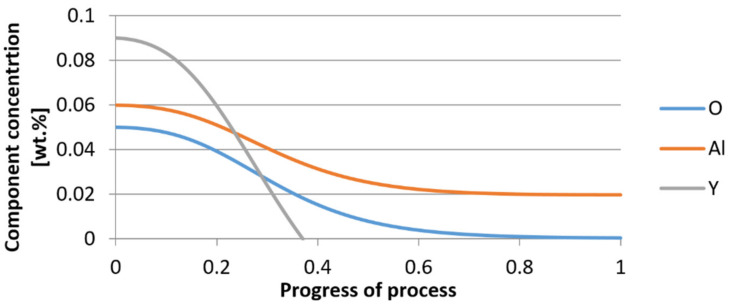
Change of components concentration during the growth of a bi-component nucleus (Al_2_O_3_–Y_2_O_3_) in steel 1 (for *r*_0_ = 5 µm).

For variant I, the following data were assumed: the number of nuclei in 1 cm^3^ of steel is 10^6^, the elemental concentration in steel: Al = 0.06%, Y = 0.09%, O = 500 ppm, equilibrium concentration of O = 0.009%, β = 1, molar volume of oxides *v*_1_ and *v*_2_ = 20 cm^3^. Aluminum and yttrium are introduced in the final step of the refining process into the pre-oxidized steel. The initial oxygen content of the steel assumed in the calculations (500 ppm) is intentionally inflated to highlight the simulation results. Figure 14, Figure 15, Figure 16, Figure 17, Figure 18 and Figure 19 shows the results of the calculations, representing the increase in nucleation radius and the molar increase in oxides *n*_1_ (Al_2_O_3_) and *n*_2_ (Y_2_O_3_) for a bi-component nucleus with an initial radius *r*_0_ = 1 µm and *r*_0_ = 5 µm.

From calculations of the growth of the bi-component oxide nucleus, it was found that at the initial stage of the process (for a process progression of about 0.4), the molar increment of the yttrium oxide and alumina phase is the same for both nuclei with an initial radius of 1 µm and also 5 µm. The greatest increase in embryo radius is also observed in this range. When yttrium reaches equilibrium content with oxygen, further growth of the nuclei takes place through the formation of alumina. The course of the line of change of the elemental proportion in liquid steel as a result of the following precipitation processes shows that yttrium is consumed for the formation of the oxide phase, and the occurring deficit of this element prevents further growth of the embryo in the form of yttrium-based precipitation.

**Figure 20:**
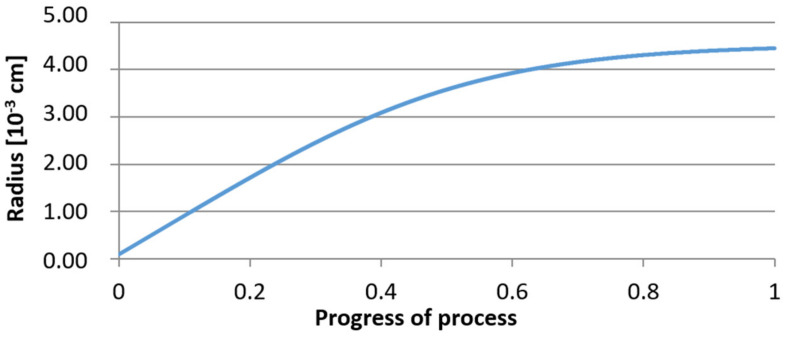
Growth of the radius of nucleus (with an initial radius *r*_0_ = 1 µm) in steel 2.

**Figure 21:**
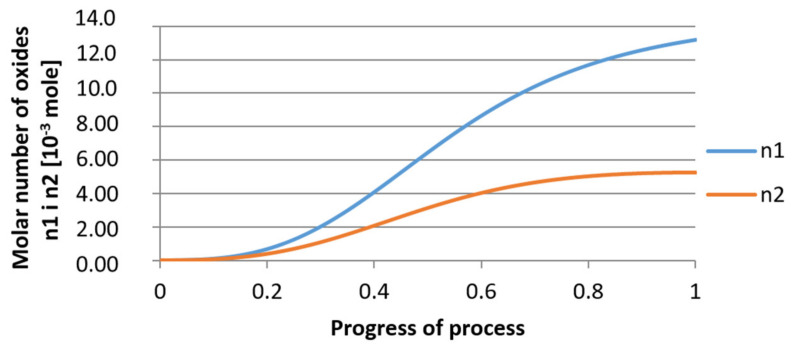
Growth of bi-component nuclei of oxides *n*_1_ (Al_2_O_3_) and *n*_2_ (Y_2_O_3_) in steel 2 (for *r*_0_ = 1 µm).

**Figure 22:**
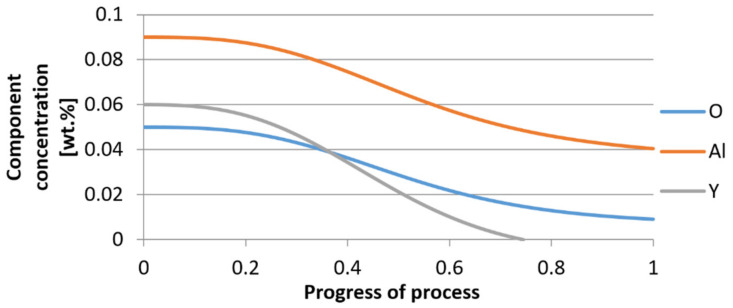
Change in component concentration during bi-component nucleus growth (Al_2_O_3_–Y_2_O_3_) in steel 2 (for *r*_0_ = 1 µm).

**Figure 23:**
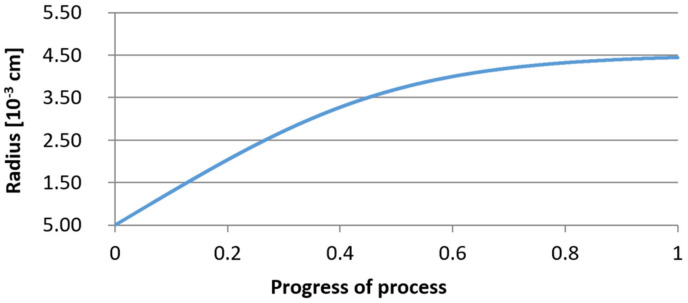
Growth of the radius of nucleus (with initial radius *r*_0_ = 5 µm) in steel 2.

**Figure 24:**
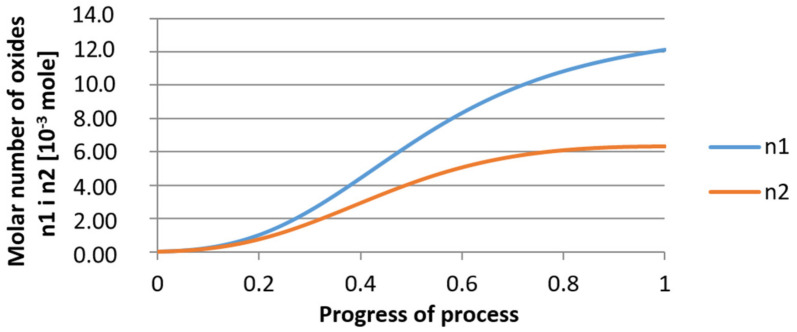
Growth of bi-component nuclei of oxides *n*_1_ (Al_2_O_3_) and *n*_2_ (Y_2_O_3_) in steel 2 (for *r*_0_ = 5 µm).

**Figure 25:**
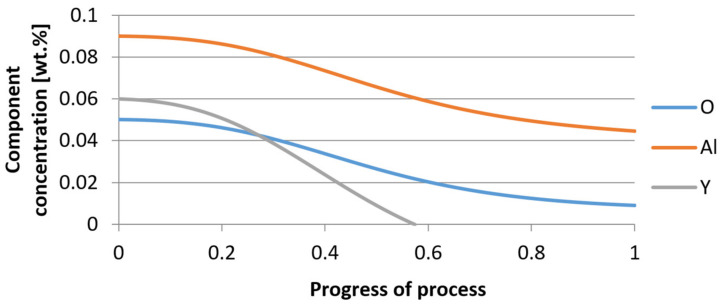
Change of component concentration during the growth of a bi-component nucleus (Al_2_O_3_–Y_2_O_3_) in steel 2 (for *r*_0_ = 5 µm).

For calculations according to variant 2, the following data were assumed: the number of germs in 1 cm^3^ of steel is 10^6^, Concentration: Al = 0.09%, Y = 0.06%, O = 500 ppm, equilibrium concentration of O = 0.009%. In Figure 20, Figure 21, Figure 22, Figure 23, Figure 24 and Figure 25, the results of calculating the radius growth of an embryo composed of oxides are presented: Al_2_O_3_ and Y_2_O_3_ for steel 2, whose chemical composition had a lower yttrium concentration (0.06%). The curves in Figure 20 and Figure 23 represent the radius growth of a bi-component nucleus with an initial radius *r*_0_ = 1 µm and *r*_0_ = 5 µm. The following figures show the molar growth of oxides *n*_1_ (Al_2_O_3_) and *n*_2_ (Y_2_O_3_) and the change in the chemical composition of liquid steel as a result of precipitation processes.

It was observed that the uniform molar growth of aluminum and yttrium oxides in the nucleus occurs only in the initial stage of the process (for about 0.2 process progress); in the latter stage, the growth occurs due to the formation of the aluminum oxide phase, with the aluminum reaching equilibrium with oxygen in the final stage (for about 0.8 process progress). The pattern of change in yttrium concentration in the steel is interesting. For a 1 µm radius nucleus, yttrium deficiency occurs at a process progression of about 0.75; and for a 5 µm radius nucleus, this phenomenon occurs much earlier. Simulation results of the growth of bi-component oxide nuclei for variants 1 and 2 showed that yttrium is a deficient element, and its rapid consumption by reaction with oxygen is due to the stoichiometry of oxide formation Y_2_O_3_ and, compared to aluminium, a much higher molar mass.

**Figure 26:**
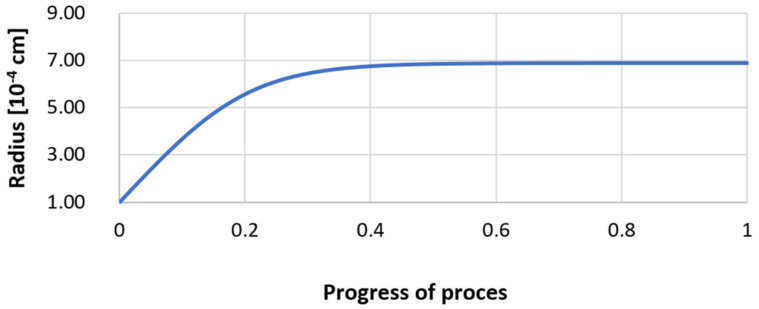
Growth of the radius of nucleus (with an initial radius *r*_0_ = 1 µm) in steel 3.

**Figure 27:**
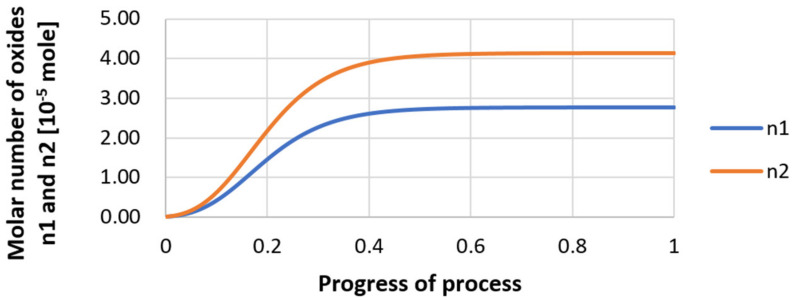
Growth of bi-component nuclei of sulfide *n*_1_ (Y_2_S_3_) and oxide *n*_2_ (Y_2_O_3_) in steel 3 (for *r*_0_ = 1 µm).

**Figure 28:**
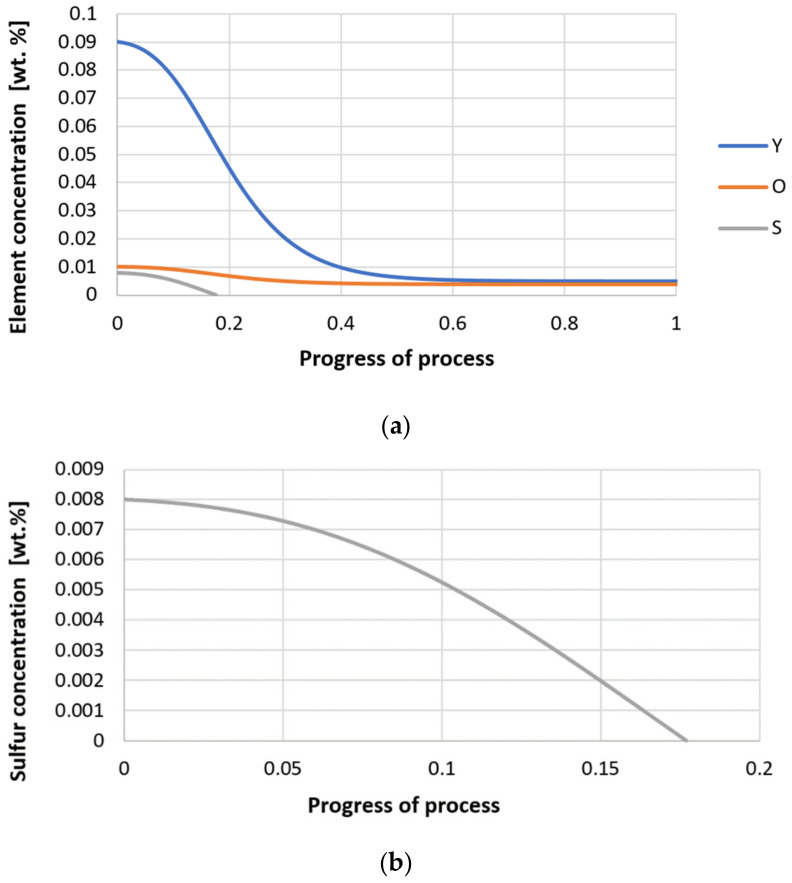
(**a**,**b**) Change of component concentration during the growth of a bi-component nucleus (Y_2_O_3_–Y_2_S_3_) in steel 3 (for *r*_0_ = 1 µm).

**Figure 29:**
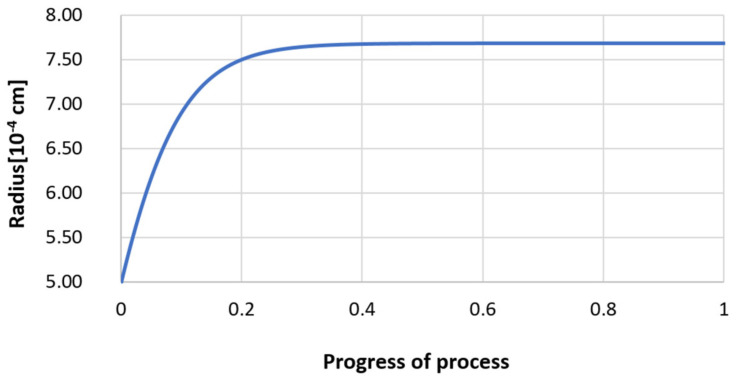
Growth of the radius of nucleus (with an initial radius *r*_0_ = 5 µm) in steel 3.

**Figure 30:**
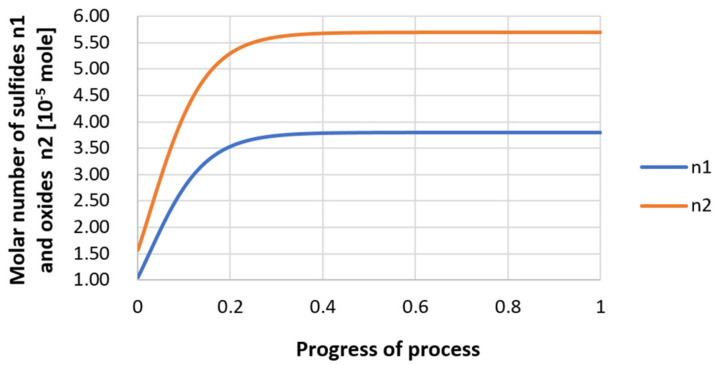
Growth of bi-component nuclei of sulfide *n*_1_ (Y_2_S_3_) and oxide *n*_2_ (Y_2_O_3_) in steel 3 (for *r*_0_ = 5 µm).

**Figure 31:**
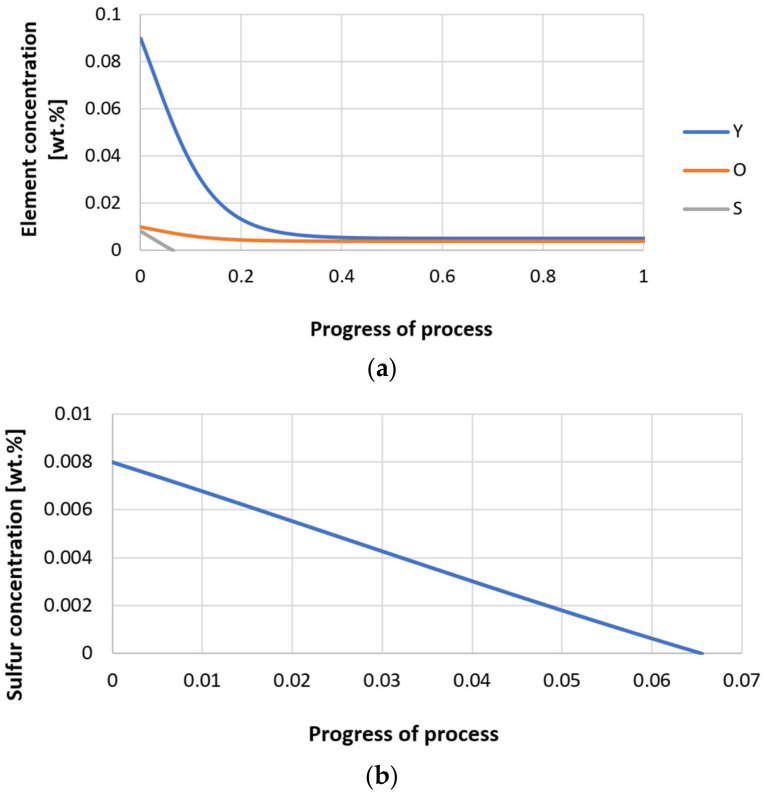
(**a**,**b**) Change of component concentration during the growth of a bi-component nucleus (Y_2_O_3_–Y_2_S_3_) in steel 3 (for *r*_0_ = 5 µm).

Next, calculations for variant 3 were performed, and the following data were used for the calculations: number of nuclei in 1 cm^3^ of steel equals to 10^6^, concentration: S = 0.008%, Y = 0.09%, O = 0.01%, equilibrium concentration for Y = 0.005%, molar volume *v*_1_ of sulfide 20 [cm^3^·mol^−1^], molar volume *v*_2_ of oxide 20 [cm^3^·mol^−1^]. Figure 26, Figure 27, Figure 28, Figure 29, Figure 30 and Figure 31 present calculation results, representing the increase in nucleus radius, the molar growth of a bi-component inclusion composed of *n*_1_ of sulfide (Y_2_S_3_) and *n*_2_ of oxide (Y_2_O_3_) for an inclusion with initial radius *r*_0_ = 1 µm and *r*_0_ = 5 µm, and a change of chemical composition of liquid steel during precipitation processes. Figure 26 and Figure 29 show the growth pattern of the radius of a bi-component nucleus. For a particle with initial radius *r*_0_ =1 µm, the radius grows mainly in the first phase of the process up to about 0.4 process progression; further increment is small and is due to yttrium reaching equilibrium concentration and sulfur deficiency. In the case of a nucleus with an initial radius of *r*_0_ = 5 µm, growth also takes place at an early stage of growth, with equilibrium establishing earlier, i.e., by about 0.2 process progression. Figure 27, Figure 28, Figure 30 and Figure 31 show the course of the change in the concentration of elements dissolved in liquid steel and the growth of the oxide and sulfide phases in the inclusion. In both cases it is observed that the calculated molar volumes of selected non-metallic inclusions and the low initial content of sulfur in the steel causes it to be consumed for the formation of the sulfide phase, whereas the inhibition of the growth of the oxide phase is due to yttrium reaching an equilibrium (v = 20 сm^3^·mol^−1^). Next, simulations were performed for steel 1 (variant 1) using empirically determined molar volumes (Table 5), assuming the initial radius of the nucleus *r*_0_ = 5 µm.

Use of empirically determined molar volumes of oxide in calculations *n*_1_ and *n*_2_ significantly changed the simulation results compared to the calculations obtained for variant 2 (where equal molar volumes were assumed for the oxides *n*_1_ and *n*_2_). It was observed that the molar growth of the binary embryo is mainly done by the formation of the alumina phase. It was found that the precipitation process causes yttrium to reach an equilibrium concentration at the end of the process (for 0.8 process progress), and there is no deficit of this element despite the lower initial concentration (0.05% Y) compared to the higher aluminum content in the steel (0.09% Al). The same series of calculations was performed for the growth of binary oxide-sulfide embryos (variant 3).

It is observed that the sulfur deficit resulting from obtaining a very low concentration causes the growth of the binary embryo to take place by the formation of the oxide phase. The calculation results obtained are similar to those obtained for variant 3 and the equal molar volumes of the oxide and sulfide phases assumed then.

The influence of the parameter β was analyzed (variant 4) and the growth process of bi-component oxide nuclei composed of Y_2_O_3_ and Al_2_O_3_ (input data for variant 1) and Y_2_S_3_ and Y_2_O_3_ (input data for variant 3) with initial radius *r*_0_ = 5 µm.

It was found that parameter β has a significant effect on the precipitation process; a significant decrease in the value of the mass penetration factor (β) visibly slows down the nucleus growth process. This phenomenon is particularly evident in the case of the growth of bi-component oxide-oxide inclusions. For conditions of refining by inert gas of a liquid metal bath in a ladle, the value of the parameter adopted for calculations β = 1 is correct. If the case of a lower parameter β = 0.5, calculation results obtained for the radius growth of oxide inclusions are very similar. A similar trend was observed for the radius growth of the sulfide-oxide nucleus.

## 4. Conclusions

Yttrium forms non-metallic inclusions in liquid steel by reacting with O and S to form a stable oxide, sulfide, and oxy-sulfide system. In the presence of other elements with high chemical affinity for oxygen and sulfur it can form complex non-metallic precipitates, as confirmed by phase diagram analysis and simulation results with the WYK_Steel program. This program proved that a population of chemically diverse precipitates concurrently appears in liquid steel with deoxidizing and modifying additives. Calculations have shown that the products of chemical reactions occurring during preoxidation contain mainly oxide precipitates, and the proportion of each phase depends on the yttrium addition. If first aluminum and second yttrium are introduced to liquid steel, the proportion of the Al_2_O_3_ phase remains on the same level, which means that yttrium losses are significant due to the formation of the oxide phase. This effect is also enhanced by the increased sulfur content in the system, which results in the formation of the yttrium sulfide phase. In turn, the formation of yttrium sulfide phase at low oxygen content eliminates the formation of manganese sulfide. Thus, in order to reduce the loss of yttrium for the formation of sulfide precipitates, the metal bath has to be completely desulfurized. The obtained calculation results presented in the form of graphs suggest that complex precipitates can be formed. This is determined by the concentrations of the components making up the liquid metal bath, whereas the amount of the inclusion is influenced by the concentration of the component, whose deficiency has been observed earlier. The chemical composition of bi-component inclusions Al_2_O_3_–Y_2_O_3_ with radius 1 and 5 µm depends on the oxygen and deoxidant contents of liquid steel. The deoxidizing ability of yttrium is much stronger than that of aluminum, so the concentration of yttrium in liquid steel will determine the nature of the inclusion growth and the final oxygen content of liquid steel. In the case of oxide-sulfide nucleus growth, the nature of the growth is determined by the presence of sulfur. The deficiency of sulfur resulting from its low concentration in liquid metal bath inhibits the growth of the sulfide phase, and further growth of the particle takes place by the formation of the oxide phase. The slowing down of this process results from the fact that yttrium reached its equilibrium concentration. In modeling the nuclei growth phenomenon, hydrodynamic conditions must be considered, which has reference to the magnitude of the coefficient β. If liquid steel was poorly mixed, the diffusion phenomena and diffusion coefficients of individual components should also be considered. Such a variant would relate to the processes occurring in the ingot mold or crystallizer during casting. The change in activity of oxides and sulfide in liquid and solid solution also needs consideration.

## Figures and Tables

**Table 1:** Standard Gibbs energy and equilibrium constants of the reaction with yttrium in the temperature range 1575–1625 °C.

Reaction	∆G° = C – Dt [J·mol^−1^]		$\log\;K=-\left(\frac{A}{T}\right)+B$		K
	$10^{-6}C$	$10^{-3}D$	$10^{-4}A$	B	**(1600 °C)**
Y_2_O_3(s)_ = 2[Y] + 3[O]	1.793	0.658	9.365	34.40	2.5 × 10^−16^
Y_2_O_2_S_(s)_ = 2[Y] + 2[O] + [S]	1.521	0.536	7.949	28.03	4.1 × 10^−15^
Y_2_S_3(s)_ = 2[Y] + 3[S]	1.171	0.441	6.119	23.10	$2.4\;\times \;10^{-10}$
YS_(s)_ = [Y] + [S]	0.321	0.091	1.677	4.74	$6.1\;\times \;10^{-5}$
YN_(s)_ = [Y] + [N]	0.391	0.150	2.044	7.86	$8.9\;\times \;10^{-4}$
YC_2(s)_ = [Y]+2[C]	1.704	0.124	0.809	6.49	5.210

**Table 2:** Variants of the models and calculations using the WYK_STAL computer program.

Variant	Input Parameters	System Properties
1-model a	1min 30 kg Al 10 min 76 kg Y 20 min 20 kg Ca	activity of the formed compound a = 1
2-model c	1min 30 kg Al 10 min 76 kg Y 20 min 20 kg Ca	metal-slag interfacial partition coefficient
3-model a	1 min 30 kg Al 10 min 30 kg Ca 20 min 76 kg Y	activity of the formed compound a = 1
4-model c	1 min 30 kg Al 10 min 30 kg Ca 20 min 76 kg Y	metal-slag interfacial partition coefficient

**Table 3:** Chemical composition of steel (wt.%).

Component	C	Mn	Si	P	N	S
wt.%	0.054	0.05	0.23	0.007	0.005	max 0.02

**Table 4:** Calculated molar volumes of selected non-metallic inclusions.

Chemical Compound	Molar Volume *v* of Precipitate (cm^3^·mole^−1^)
Y_2_O_3_	45.07
Y_2_S_3_	70.80
Y_2_O_2_S	49.39
Al_2_O_3_	25.82

**Table 5:** Calculated molar volumes of selected non-metallic inclusions.

Chemical Compound	Molar Volume *v* of Precipitate (cm^3^·mol^−1^)
Y_2_O_3_	45.07
Y_2_S_3_	70.80
Y_2_O_2_S	49.39
Al_2_O_3_	25.82

## Data Availability

Data are contained within the article.
